# Nanovortex‐Driven All‐Dielectric Optical Diffusion Boosting and Sorting Concept for Lab‐on‐a‐Chip Platforms

**DOI:** 10.1002/advs.201903049

**Published:** 2020-04-24

**Authors:** Adrià Canós Valero, Denis Kislov, Egor A. Gurvitz, Hadi K. Shamkhi, Alexander A. Pavlov, Dmitrii Redka, Sergey Yankin, Pavel Zemánek, Alexander S. Shalin

**Affiliations:** ^1^ ITMO University Kronverksky prospect 49 St. Petersburg 197101 Russia; ^2^ Institute of Nanotechnology of Microelectronics of the Russian Academy of Sciences (INME RAS) Nagatinskaya Street, House 16A, Building 11 Moscow 119991 Russia; ^3^ Electrotechnical University “LETI” (ETU) 5 Prof. Popova Street Saint Petersburg 197376 Russia; ^4^ LLC COMSOL Bolshaya Sadovaya St. 10 Moscow 123001 Russia; ^5^ Czech Academy of Sciences Institute of Scientific Instruments Královopolská 147 Brno 612 64 Czech Republic

**Keywords:** all‐dielectric nanophotonics, lab‐on‐a‐chip platforms, nanofluidics, optomechanical manipulations, spin‐orbit couplings

## Abstract

The ever‐growing field of microfluidics requires precise and flexible control over fluid flows at reduced scales. Current constraints demand a variety of controllable components to carry out several operations inside microchambers and microreactors. In this context, brand‐new nanophotonic approaches can significantly enhance existing capabilities providing unique functionalities via finely tuned light−matter interactions. A concept is proposed, featuring dual on‐chip functionality: boosted optically driven diffusion and nanoparticle sorting. High‐index dielectric nanoantennae is specially designed to ensure strongly enhanced spin−orbit angular momentum transfer from a laser beam to the scattered field. Hence, subwavelength optical nanovortices emerge driving spiral motion of plasmonic nanoparticles via the interplay between curl−spin optical forces and radiation pressure. The nanovortex size is an order of magnitude smaller than that provided by conventional beam‐based approaches. The nanoparticles mediate nanoconfined fluid motion enabling moving‐part‐free nanomixing inside a microchamber. Moreover, exploiting the nontrivial size dependence of the curled optical forces makes it possible to achieve precise nanoscale sorting of gold nanoparticles, demanded for on‐chip separation and filtering. Altogether, a versatile platform is introduced for further miniaturization of moving‐part‐free, optically driven microfluidic chips for fast chemical analysis, emulsion preparation, or chemical gradient generation with light‐controlled navigation of nanoparticles, viruses or biomolecules.

## Graphical abstract

The power of the spin−orbit coupling effect in all‐dielectric nanophotonics is harnessed to propose a novel, all‐optical nanomixing and sorting device potentially integrable in lab‐on‐a‐chip platforms. Specifically, under simple plane wave illumination, a dielectric structure with carefully tailored optical near fields is shown to induce nanofluid vortices, effectively boosting diffusion of admixtures.

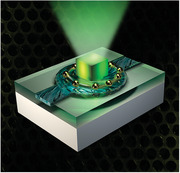

## 1 Introduction

Micro‐optofluidics represents one of the most promising and fast growing fields in current state‐of‐the‐art science and engineering.^[^ ^1^, ^2^, ^3^, ^4^, ^5^, ^6^, ^7^ ^]^ In particular, the control of fluid flows in microsized channels plays an essential role for applications ranging from the transport of reduced amounts of hazardous or costly substances and DNA biochip technology, to miniaturized analytical and synthetic chemistry.^[^ ^8^, ^9^, ^10^, ^11^, ^12^ ^]^

The multidisciplinary nature of microfluidics has brought together seemingly unrelated fields, such as electrical and mechanical engineering, biology, chemistry, and optics. For example, in the context of chemical engineering, the utilization of distributed microreactors working in parallel can enhance production significantly and facilitates the design of new products.^[^ ^13^, ^14^ ^]^ However, slow mixing processes constitute a bottleneck that restricts reaction processes, especially when the desired reaction rate is high.^[^ ^15^, ^16^ ^]^ For this purpose, fast mixing is highly required to avoid the reactive process being delayed by this critical step, and to reduce potential side products.^[^ ^16^ ^]^

Given the low Reynolds numbers at which fluid flow occurs in microreactors, fluid mixing represents a significant challenge.^[^ ^4^, ^17^, ^18^ ^]^ In the most conventional situation where only passive mixing happens, the main driving mechanism corresponds to diffusion (Brownian motion^[^ ^4^ ^]^), implying mixing to take place at a very low rate. Consequently, the effective distance that the molecules of a fluid need to travel in a mixer before interacting with another fluid (i.e., the mixing length) becomes restrictively long.^[^ ^18^ ^]^ Passive mixers depend solely on decreasing the mixing length by optimizing the flow channel geometry in order to facilitate diffusion.^[^ ^4^, ^19^ ^]^ In contrast, active schemes rely on external sources injecting energy into the flow in order to accelerate mixing and diffusion processes and drastically decrease the mixing lengths.^[^ ^17^, ^20^ ^]^

Most early studies related to micromixers have been focused on the passive type. Conversely, despite their higher cost and complex fabrication methods, the enhanced efficiency of active micromixers with respect to passive ones has drawn the attention of the scientific community in the recent years.^[^ ^17^ ^]^ Because of the power and size constraints involved in microfluidics, research efforts have been focused on the utilization of mixing principles not involving moving mechanical parts such as surface tension‐driven flows,^[^ ^21^ ^]^ ultrasound and acoustically induced vibrations,^[^ ^22^, ^23^ ^]^ and electro‐ and magnetohydrodynamic action.^[^ ^18^, ^24^ ^]^

Given the small operation scales of microfluidics, micron‐scale focusing of laser beams, as well as different types of light−matter interactions make possible to provide sufficiently strong optical forces to propel particles,^[^ ^25^, ^26^, ^27^ ^]^ sort objects according to their size or optical properties,^[^ ^5^, ^28^, ^29^, ^30^, ^31^ ^]^ or self‐arrange colloidal particles into optically bound structures.^[^ ^25^, ^26^, ^27^, ^32^, ^33^ ^]^ Nowadays the additional degrees of freedom offered by complex shaping of laser beams^[^ ^34^, ^35^, ^36^, ^37^, ^38^ ^]^ have made possible the manipulation and trapping of large amounts of microparticles.^[^ ^39^ ^]^ In particular, they allow to create optical vortices with helical phase front (e.g., Laguerre−Gaussian or higher‐order Bessel beams) carrying both linear and angular momentum.^[^ ^40^, ^41^ ^]^ When such an optical microvortex is scattered by particles, it induces an optical torque on them leading to their orbital motion around the focus of the laser beam.^[^ ^42^ ^]^ Due to the angular momentum conservation, elastic scattering of a circularly polarized beam possessing spin−angular momentum (SAM),^[^ ^43^ ^]^ by optically anisotropic^[^ ^44^, ^45^, ^46^ ^]^ or nonspherical objects^[^ ^47^, ^48^ ^]^ leads to their spinning around the direction of propagation of the incident illumination. Combining both types of optical angular momentum leads to complex spin−orbital interaction^[^ ^41^, ^49^ ^]^ and novel interesting phenomenon, e.g. detection of spin forces.^[^ ^50^, ^51^ ^]^

At the nanoscale, metal‐based plasmonics dominates and provides exciting means for trapping and manipulating nano objects.^[^ ^52^, ^53^, ^54^, ^55^ ^]^ On the other hand, the recently growing field of all‐dielectric nanophotonics^[^ ^56^ ^]^ presents itself as a promising alternative for the integration of optomechanical concepts in microfluidic devices. Properly designed dielectric nanostructures with finely tuned Mie resonant response provide the means for tailoring electric and magnetic components of the scattered light.^[^ ^56^, ^57^, ^58^ ^]^ They allow to obtain strong near fields, which induce substantial optical forces acting upon other subwavelength scatterers dispersed in the medium surrounding the nanostructure.^[^ ^59^, ^60^, ^61^, ^62^, ^63^ ^]^

**Figure 1:**
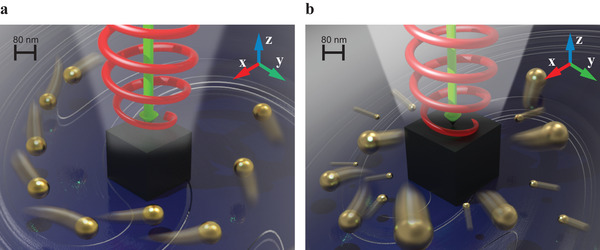
An artistic view of the proposed active nanomixing scheme (left) and radial separation of nanoparticles (right). a) A silicon nanocube submerged in a water solution is illuminated by a circularly polarized laser beam coming from the top. The scattered field carries a nonzero tangential component of the pointing vector in the *xy* plane, which induces nonzero orbital angular momentum in the negative *z* direction. The same effect causes the spiral motion of Au nanoparticles around the nanocube. Viscous friction between the moving nanoparticles and the fluid gives rise to convective fluid motion and enhances fluid mixing. b) Sorting concept. Nanoparticles of different sizes having opposite signs of the real part of polarizability are radially displaced in opposite directions—the smaller ones move toward the nanocube, while larger ones move away from it.

In this work, we focus on the conversion of SAM of an incident circularly polarized plane wave into orbital angular momentum (OAM)^[^ ^64^, ^65^ ^]^ of the scattered field mediated by a specially designed nanostructure constituted of a realistic high refractive index material (silicon). In contrast to the above‐mentioned methods, the optical vortex field created in this way is very localized, only reaching a few hundreds of nanometers in diameter. Moreover, the induced optical forces are strong enough to propel gold (Au) nanoparticles of particular sizes along spiral trajectories around the nanostructure. Based on the latter effect, we propose a novel method for mixing fluid in nanovolumes mediated by chemically inert Au nanoparticles (see **Figure** **1**a). In addition, we take advantage of the size sensitivity of the Au polarizability to achieve light‐mediated nanoparticle separation by means of the same geometrical configuration (see Figure 1b). The subwavelength size of the investigated optical nanovortex greatly enhances the length scale of interaction in comparison to the more conventional approaches involving Bessel beams,^[^ ^66^, ^67^ ^]^ opening new directions in light−matter interaction via light angular momentum exchange. We believe that the proposed simple geometry for optically driven diffusion boosting and nanoparticle sorting is of high interest for a plethora of applications in microfluidics and lab‐on‐a‐chip devices.

## 2 Formation of an Optical Nanovortex

The spiral motion of nano‐objects in an optical nanovortex driven by an out‐of‐plane light source (Figure 1a) requires, on the one hand, efficient transformation of SAM of light to in‐plane OAM of the highly confined near fields of the nanocube, which, in turn, should be transferred to the surrounding nanoparticles. Therefore, as a first constraint, sufficient in‐plane scattering from the nanocube should take place. This urgent functionality could be enabled, in particular, by the recently observed Transverse Kerker Effect^[^ ^68^ ^]^ allowing for lateral‐scattering only. On the other hand, azimuthal forces arising due to helicity inhomogeneities in the scattered near field (curl−spin forces^[^ ^69^ ^]^) may also enable rotational motion. Hereinafter, we optimize both effects taking into account that we, actually, do not require the total suppression of forward and backward scattering as in,^[^ ^68^ ^]^ and, therefore, we can tune the parameters in order to obtain an enhanced optical subwavelength vortex.

**Figure 2:**
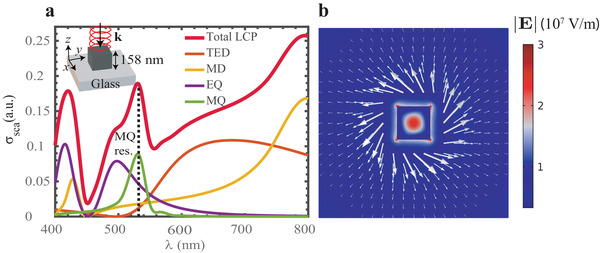
a) Cartesian multipole decomposition of the scattering cross‐section of the silicon cube deposited on a glass substrate, centered at the origin of the coordinate system; the cube is illuminated by a left‐hand circularly polarized plane wave propagating against the *z*‐axis. The geometry is illustrated in the top inset and the ambient medium is water. The dashed black line indicates the position of the resonant MQ mode (green laser, 532 nm). The total scattered power with incident LCP illumination (total LCP) is fully reconstructed as the sum of contributions of individual multipoles, respectively, the total electric dipole (TED), magnetic dipole (MD), electric quadrupole (EQ), and the MQ. b) Colorplot denotes the norm of the total electric field at 532 nm wavelength in the transverse *x−y* plane at *z* = 70 nm. The arrows indicate direction and their lengths illustrate the relative size of the transverse part of the scattered Poynting vector. The parameters of the cube are given in Table S1, Supporting Information.

A cubical Si nanoantenna with refractive index *n* ≈ 4 (e.g., silicon at the visible range) and edge 158 nm is illuminated by a circularly polarized plane wave propagating along the negative *z*‐axis (see inset of **Figure** **2**a). For such an incident field one can calculate the angular momentum flux density using the expressions for paraxial waves.^[^ ^70^ ^]^ Since the wave carries no OAM, the total angular momentum flux density is entirely given by the SAM flux density (see the Supporting Information):

(1) $$J_{z}^{inc}=\sigma \frac{I_{0}}{\omega }$$

where the wave helicity σ takes the values of +1 and −1 for left and right‐circular polarization, respectively, and *I*_0_ is the incident light intensity. Since the nanostructure has negligible losses, the total angular momentum of the incident and scattered light is conserved. This conservation law for the total angular momentum implies that part of the incident SAM, given by Equation (1), is transferred to both SAM and OAM of the scattered field leading to spin−orbit coupling phenomena.

The transverse components of the Poynting vector in the field scattered by the nanocube display similar rotating features as for chiral scatterers such as helices or gammadion‐like structures,^[^ ^71^, ^72^, ^73^ ^]^ however the fabrication process of the first is much less complex and no chirality is required. In order to gain better physical insight, we consider the multipole decomposition for the scattering cross‐section of the nanocube deposited over a glass substrate (Figure 2a) and illuminated with LCP light in a water host environment (refractive index *n_m_* = 1.335). A careful analysis reveals the most pronounced vortex‐like energy flux arises at the magnetic quadrupole (MQ) resonance (Figures 2a,b). Moreover, varying the size of the nanocube, we are able to modify the multipolar response of the resonator in order to position the MQ resonance at the vacuum wavelength 532 nm (399 nm in water) corresponding to the most convenient green laser source (see Table S1, Supporting Information, for the relevant parameters). Hereinafter we will proceed with these values, however, we should note that an even more enhanced MQ response can be obtained in a free space environment (see the Supporting Information).

The MQ mode presents a high signal‐to‐noise‐ratio with respect to the other leading multipoles; the magnetic dipole is almost one order of magnitude smaller and the electric quadrupole is out of resonance. The electric dipole radiation is also strongly suppressed by an anapole state in the vicinity of the MQ resonant frequency. Thus, well‐pronounced MQ fields can be obtained driving the vorticity of the Poynting vector^[^ ^74^ ^]^ (Figure 2b) (see also the Supporting Information).

While the numerical results shown in Figure 2 provide a clear link between the enhanced transfer of incident field SAM to scattered field OAM at the MQ resonance, a complete physical picture requires a deeper theoretical insight on the behavior of the fields produced by the MQ mode under the prescribed illumination. For that purpose, we derive in the Supporting Information the expression for the power extracted by the nanocube (referred to as the extinction power,^[^ ^75^ ^]^ *P*_ext_) from a circular plane wave propagating against the *z*‐axis

(2) $$P_{\text{ext}}=-\frac{E_{0}k_{0}^{2}}{4}\text{Re}\left\{i\sigma M_{zx}+M_{zy}\right\}$$

where *M_ij_* is *ij*‐th component of the MQ tensor. The center of the nanocube is placed at the origin of the coordinate system, with the *x*,*y* and *z*‐axis oriented perpendicular to its sides. Taking into account symmetry, we obtain the following nonzero tangential component $S_{\phi }^{s}$ ^[^ ^76^ ^]^ of the scattered Poynting vector:

(3) $$S_{\phi }^{s}=\sigma \frac{3{\left|M_{zx}\right|}^{2}}{16\pi^{2}c\mu_{0}}\frac{\left(9+3r^{2}k_{0}^{2}+r^{4}k_{0}^{4}\right)}{k_{0}r^{7}}\cos{\left(\theta \right)}^{2}\sin\left(\theta \right)$$

where θ is the polar angle in spherical coordinates, and *r* = |**r**|. In the *x−y* plane θ = π/2 and $S_{\phi }^{s}=0$.

**Figure 3:**
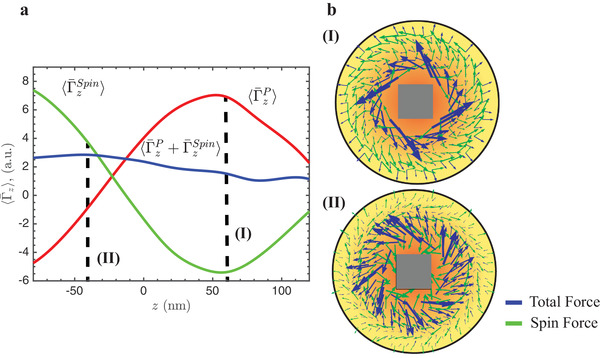
a) Optical torque affecting absorbing 40 nm radius nanoparticles with *n* ∼ 2*i* in the near field of the MQ resonance (λ = 532  nm). The time‐averaged spin ($\Gamma_{z}^{Spin}$) and radiation pressure ($\Gamma_{z}^{P}$) contributions have been spatially averaged (〈〉) in parallel *x−y* planes in the near field along the height of the cube (*z*‐axis). b) Transverse cuts at *z* = 60 nm (I) and *z* = −40 nm (II) showing the vector field distributions of total and curl‐spin forces around the cube.

The intuitive physical picture is as follows: during an oscillation period, the incident circularly polarized electric field gradually changes its polarization between the *x*‐ and y‐axis, consequently, the components of the excited MQ tensor oscillate accordingly. In analogy with a rotating electric dipole,^[^ ^77^ ^]^ Equation (2) shows that the scattered near‐field at the MQ resonance can be obtained as a superposition of the fields generated by *M_zx_* and a *πσ*/2 delayed *M_zy_* component with equal amplitudes. The *total* Poynting vector outside the scatterer, however, also includes an interference term between the scattered electromagnetic field and the incident one, which leads to nonnegligible curl in the *x*−*y* plane. This is indeed what is observed in the numerical simulations (see **Figure** **3**a, where $\Gamma_{z}^{P}$ is proportional to 〈*S*_ϕ_〉). Substituting the scattered Poynting vector in Equation (S2), Supporting Information, the time‐averaged scattered angular momentum density component in the *z*‐axis *J_z_* can be determined as

(4) $$J_{z}=\sigma \left|\frac{r}{c}S_{\phi }^{s}\right|$$

Comparing Equation (4) with Equation (1) provides direct evidence that SAM from the incident wave has been transferred to the scattered field giving rise to the optical vortex shown in Figure 2b. Moreover, since *J_z_* depends on the choice of origin of the coordinate system^[^ ^78^ ^]^ it can be directly correlated with the extrinsic OAM of the scattered field. Further inspection of Equations (3) and (4) also show that the tangential component of the Poynting vector as well as the angular momentum scale quadratically with the amplitude of the MQ moment enhancing the field vorticity at the MQ resonance. Since the angular momentum scales as *r* ^−*n*^ (where *n* is a positive integer) in the near field, the vorticity of the Poynting vector is very high close to the particle, but decreases very fast going away from it, as confirmed in Figure 2b. The latter has very important consequences regarding the optical forces governing the motion of plasmonic nanoparticles under the influence of such a field, as mentioned below. Additional analytical and numerical proofs linking the nature of the optical vortex formation to MQ scattering can be found in the Supporting Information.

In this section, we have described the excitation of the MQ mode and proposed an intuitive physical picture explaining the multipolar origin of the tangential component of the Poynting vector giving rise to an optical vortex in the near field of the nanocube.

## 3 Optical Nanovortex‐Mediated Forces and Torques

We can now proceed to study the effect of the scattered field on small (dipolar) particles. The time‐averaged optical force 〈**F**_*o*_〉 acting upon such a nanoparticle in the optical nanovortex can be written as^[^ ^79^ ^]^

(5) $$\left\langle F_{o}\right\rangle =\frac{\alpha^{\prime }}{4}\nabla (E^{*}\cdot E)+\frac{ck_{0}\alpha^{\prime \prime }}{\varepsilon_{0}}\left(\frac{1}{c^{2}}\left\langle S\right\rangle +\nabla \times \left\langle L_{s}\right\rangle \right)$$

where *n_m_* is the refractive index of the host medium, **E** is the sum of the incident and scattered (by the nanocube) electric fields, 〈**L**_*s*_〉 is the average SAM flux density (see the Supporting Information), and α ′and α ″ are the real and imaginary parts of the particle dipole polarizability, respectively. The first term on the right‐hand side of Equation (5) corresponds to conservative (curl‐free) gradient optical forces, which for positive α ′ drag the nanoparticle toward the region of maximal field intensity. The terms in round brackets describe nonconservative or “scattering” optical forces, hereinafter noted as 〈**F**_*sc*_〉. The latter receives contributions from the total Poynting vector 〈**S**〉 and the electric field contribution to the SAM flux density 〈**L**_*s*_〉.^[^ ^79^ ^]^ A MQ mode corresponds to an object of well‐defined parity, i.e., a transverse electric (TE) multipole. At the resonance, pure electric or magnetic multipoles strongly break electromagnetic duality, and, consequently, do not present a well‐defined helicity.^[^ ^74^, ^80^ ^]^ This effect manifests itself strongly in the near field,^[^ ^69^ ^]^ and implies that the SAM flux density, which is linked to the helicity density,^[^ ^74^ ^]^ features a nonuniform spatial distribution. Therefore, the second term in 〈**F**_*sc*_〉 acknowledged as the curl−spin force^[^ ^69^ ^]^ is not only nonnegligible but also plays an essential role in the dynamics of nanoparticles.

Importantly, due to spin−orbit coupling, the particles will experience an orbital torque oriented along the *z*‐axis, Γ_*z*_, confined in a subwavelength region and directly proportional to the azimuthal component of 〈**F**_*sc*_〉 (see the Supporting Information for the exact expression). The latter depends on the azimuthal components of the time‐averaged Poynting vector and the near‐field curl−spin forces, as well as on the optical response of the particles themselves (α ″).

Interestingly, in the case of the MQ, it is possible to show that the contribution of the scattered field to the curl−spin force only has an azimuthal component, i.e., it only induces orbital motion. This result is general to any magnetic (TE) multipole field. The interference with the incident illumination leads, however, to important radial and polar components (see Figure 3).

Currently, very few groups^[^ ^69^, ^81^ ^]^ have investigated optical fields where the effect of spin−curl forces can be visibly appreciated in the dynamics of moving nanoparticles. In contrast, our calculations directly prove that both the spin force and radiation pressure contribute to the induced optical torque in the vicinity of the dielectric cube.

In Figure 3a, we show the optical torque experienced in the near field by an arbitrary absorbing 40 nm radius spherical nanoparticle with *n* ≈ 2*i* calculated with Equation S19, Supporting Information, and averaged over several circular rings on parallel transverse planes (perpendicular to the incident propagation direction). Remarkably, particles whose centers of mass are located at different heights experience different contributions from the curl−spin ($\Gamma_{z}^{Spin}$) and radiation pressure ($\Gamma_{z}^{P}$) torques, as can be visually appreciated in the force field plots shown in Figure 3b. Moreover, both contributions can be opposite to the helicity of the incident wave if considered separately, but the total scattering force remains helicity locked—a result convenient for our purposes (see Section 5). It is worth noting that $\Gamma_{z}^{P}$ is nonzero at *z* = 0, contrarily to what one might initially expect from Equation (3), but we once more emphasize that the total Poynting vector entering in Equation S19, Supporting Information, includes an additional interference term between the incident and scattered field yielding a small azimuthal component. As a consequence, the zero of $\Gamma_{z}^{P}$ is displaced to a lower *z* coordinate.

In our setup, the particles are initially pushed toward the glass substrate by the incident beam intensity, where they experience a combination of radiation pressure and curl−spin torques (Figure 3b(II)).

Here, we have introduced analytical expressions for the optical forces and torques induced on dipolar absorbing nanoparticles, which allowed us to unambiguously distinguish the contributions of the radiation pressure and the spin forces. The numerical calculations presented in Figure 3 demonstrate that both the effects mediate the strongly confined (subwavelength) particle rotation with respect to the *z*‐axis (i.e., the direction of the propagation of the incident wave).

## 4 Nanoparticle Dynamics in the Optical Nanovortex

We now turn our attention toward the potential applicability of the considered effect as a mixing method for microfluidic reactors. In order to illustrate the concept, we consider the water around the nanocube contains a dilute solution of chemically inert, biologically compatible nanoparticles. The dynamics of the latter will be affected by the optical forces arising due to the interaction with the cube's scattered field together with the Brownian and viscous drag forces induced in the fluid. The obvious and most convenient candidates to act as mixing mediators are Au nanoparticles, because they would not interact with the chemical and/or biological compounds dissolved in the solutions and are utilized in a broad range of microfluidics applications.^[^ ^82^, ^83^ ^]^

In order to increase the mechanical orbital torque transferred to the Au nanoparticles and to prevent them from sticking to the walls of the nanocube due to attractive gradient forces, the ratio 〈**F**_*sc*_〉/〈**F**_*o*_〉 should be maximized. For high enough ratios, scattering forces govern the nanoparticle dynamics, causing them to undergo spiral paths around the nanocube and act as stirrers enhancing convective fluid motion and thus diffusive mixing of any admixtures present in the water solution.

The scattering force can be a leading force acting upon the nanoparticle only if the real part of the nanoparticle polarizability is negligible in contrast to the imaginary one (see Equation (5). For simplicity, we assume a spherical shape so that their dipole polarizability can be evaluated analytically with the exact Mie theory formulae by the method described elsewhere:^[^ ^84^, ^85^, ^86^ ^]^

(6) $$\alpha \left(k_{d},R_{p}\right)=i\frac{6\pi \varepsilon_{0}n_{m}^{2}}{k_{d}^{3}}a_{1}\left(m,k_{d}R_{p}\right)$$

where *k_d_* is the wavenumber in water. *a*_1_ denotes the first electric Mie coefficient,^[^ ^75^ ^]^ which depends on the refractive index contrast between the particle and the medium $m=\sqrt{\varepsilon_{Au}(\omega )}\text{/}n_{m}$ and the dimensionless parameter *k_d_R_p_*, where *R_p_* is the nanoparticle radius. Details on the model for the permittivity of the nanoparticles ε_*Au*_ with appropriate size‐dependent corrections are given in the Supporting Information. Combining Equations (5) and (6) allows for calculating the optical forces on nanoparticles with sizes even beyond the Rayleigh limit (see, e.g., the discussion in refs. ^[^ ^58^, ^87^ ^]^).

**Figure 4:**
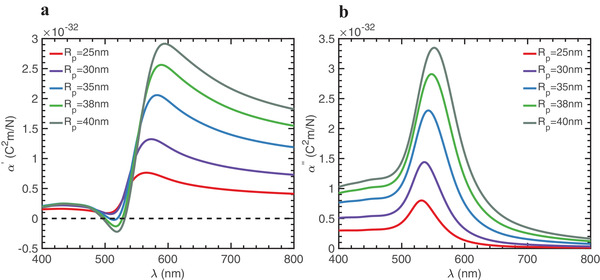
a) Real and b) imaginary parts of the dipole polarizability calculated from Equation (6) for Au nanoparticles of different sizes that are dispersed in water. The excitation wavelengths correspond to those in free space. For nanospheres with radius *R_p_* ≥ 35 nm, the real part can be equal to zero while the imaginary part is enhanced.

**Figure** **4** shows the real and imaginary parts of the polarizability for Au particles of different sizes dispersed in water. We exploit the fact that, in the vicinity of the plasmon resonance, nanoparticles with *R_p_* ≥ 35 nm can fulfill the condition α ′ = 0 with enhanced values of α ′′. For example, for particles with *R_p_* = 40 nm, the full suppression of the gradient force occurs at 500 and 530 nm (see Figure 4a). Consequently, only scattering forces are allowed for them, and the ratio 〈**F**_*sc*_〉/〈**F**_*o*_〉 is maximized. Moreover, these wavelengths are in a close proximity to the chosen green source (532 nm).

Large Au nanoparticles with *R_p_* ≥ 35 nm could be considered to break the limits of the electric dipole approximation assumed in Equation (5). To prove its validity for quantitative calculations of the optical forces, we have compared our results with exact numerical computations via integrating the Maxwell stress tensor over a 40 nm radius Au nanoparticle and obtained very good agreement (see Supporting Information). Moreover, the electric field distribution in the system plotted in Figure S3B−D, Supporting Information, shows negligible perturbations in the presence of a Au nanoparticle with no backscattering, further confirming the validity of the involved approximations.

In order to determine the trajectories of the Au nanoparticles in water, we assume the system has reached stationary equilibrium in the *z* direction, due to the compensation of the *z* component of the incident radiation pressure in the presence of the glass substrate. Therefore, the trajectories can be treated as two‐dimensional, localized only in the transverse *x*−*y* plane.

Considering scattering force 〈**F**_*sc*_〉, viscous drag force **F**_*D*_, commonly given by Stokes law, and stochastic Brownian (thermally activated) forces **F**_*B*_ acting on the nanoparticle of mass *m_p_*, the Langevin equation of motion can be written in the following form:

(7) $$\left\langle F_{sc}\right\rangle +F_{B}+F_{D}=m_{p}\;{\ddot{r}}_{p}$$

where ${\ddot{r}}_{p}$ is the particle instantaneous acceleration vector. Once the scattering force distribution is determined, Equation (7) can be solved in Comsol Multiphysics.^[^ ^88^ ^]^

To accurately reproduce the motion of the nanoparticles while restricting ourselves to a two‐dimensional analysis, the term **F**_*D*_ needs to take into account the additional drag induced by the walls of the nanocube and the glass substrate. To address this issue, we derive in the Supporting Information a modified Stokes law where the dynamic viscosity of water is replaced by a position‐dependent effective viscosity tensor $\bar{\bar{\mu }}$, which we implement in our calculations. When the nanoparticles are sufficiently far away from the nanocube, we demonstrate that our expression corresponds to the well‐known Faxen corrections for viscous flow over a planar surface^[^ ^89^, ^90^, ^91^ ^]^ (see Section 9 in the Supporting Information). We assume the system to be at ambient temperature.

The parameters for the simulations are given in Tables S1 and S2, Supporting Information.

**Figure 5:**
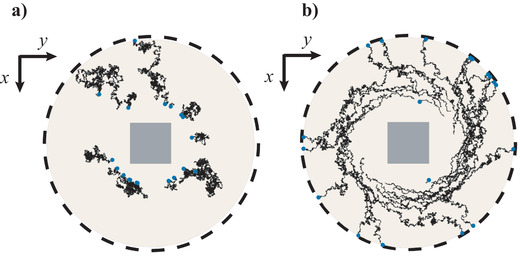
Trajectories of Au nanoparticles of 40 nm radius during 0.1 ms of simulations. Illumination wavelength in vacuum was 532 nm (in water 399 nm). a) No incident illumination, only Brownian motion and drag forces act on the particles; b) The nanocube is illuminated with a circularly polarized light, and the optical force contributes significantly. The Au nanoparticles spirally move around the cube. The figures are scaled to the length of the cube side equal to 158 nm.

We consider that Au nanoparticles of 40 nm radius are uniformly distributed around the Si nanocube. Their trajectories during a simulation time of 0.1 ms are shown in **Figure** **5**. If the nanocube is not illuminated (Figure 5a), Brownian motion induces random displacements of the nanoparticles independently on their position in the simulation domain. Conversely, when the system is illuminated with circularly polarized light with intensities about 50–80 mW µm^−2^ (corresponding to typical values utilized in conventional optical trapping schemes^[^ ^92^ ^]^), mechanical orbital torque is transferred to the Au nanoparticles and drives them along spiral trajectories (Figure 5b).

**Figure 6:**
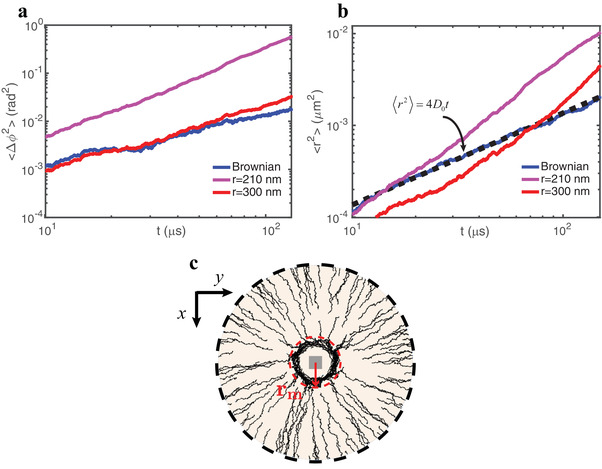
a,b) Log−log plots of MSAD and MSD characterizing the diffusional motion of Au nanoparticles under the influence of the optical near fields and allowing the determination of the range of action of the nanovortex, *r*_m_; a) Calculated MSAD averaged over 100 Au nanoparticles of 40 nm radius, when the cube is not illuminated (blue), and under LCP illumination at a distance from the center of the nanocube much lower than *r*_m_ (shown in (c)) – violet and at *r*_m_ – red. b) Averaged MSD in the same conditions as in (a). Dashed line corresponds to the fit with Einstein's relation. c) Trajectories of 40 nm radius Au nanoparticles inside and outside the nanovortex region delimited by *r*_m_. The red dashed circle delimits the effective radius *r*_m_, which determines the range of action of the tangential optical forces. The optical nanomixing effect is thus achievable at the positions lying inside *r*_m_.

Equation (3) reveals that $〈S_{\phi }^{s}〉$ becomes negligible far from the nanocube. Furthermore, the numerical simulations show that the curl−spin force has no longer a significant effect. Consequently, Brownian motion and conventional radiation pressure start to dominate the dynamics (see **Figure** **6**c).

In order to characterize the trajectories of the nanoparticles inside the vortex and the enhancement of their diffusional motion, we calculated their mean‐squared angular displacements (MSAD, 〈Δϕ^2^〉 in Figure 6a), with respect to the center of the nanocube, as explained in Section 10 of the Supporting Information.

Due to the persistent orbital torque inside the vortex, the nanoparticles are actively rotating, as shown by the remarkable increase in the azimuthal angle as a function of time. The MSAD curves can be fitted with equations of the form $〈\phi {\left(t\right)}^{2}〉=D_{R}^{eff}t+\omega_{avg}^{2}t^{2}$, where $D_{R}^{eff}$ is an effective rotational diffusion coefficient and ω_*avg*_ is the average angular speed. This analysis leads, as expected, to ω_*avg*_ = 0 for the case of passive (Brownian) diffusion, and $\omega_{avg}=0.37\;\deg\;\mu {\text{s}}^{-1}$, $0.08\;\;\deg\;\mu {\text{s}}^{-1}$ for particles placed, respectively, at 210 and 300 nm from the center of the nanocube. The results confirm that, in the near field of the nanocube, strong rotational motion can be achieved, and the nanoparticles enter a “superdiffusive” regime. Based on the previous information, we introduce the effective radius *r_m_* which specifies the area of influence of the optical nanovortex (red dashed circle in Figure 6c). For *r* ≤ *r_m_*, the majority of the Au nanoparticles circulate around the nanocube, and the dielectric nanocube acts as an effective optical drive for convective stirring of the fluid around it. The translational mean‐squared displacements of the nanoparticles (MSD, 〈*r* ^2^〉 in Figure 6b, see Section 10 in the Supporting Information), also show an important increase compared to thermal diffusion in the absence of laser illumination. We remark that the Brownian MSDs are in full agreement with the Einstein−Smoluchowski relation^[^ ^93^ ^]^ 〈*r*(*t*)^2^〉 = 4*D*_0_ *t*, where *D*_0_ is the diffusion coefficient calculated for spherical particles as *D*_0_ = *k_B_T*/6*πμ*_*xx*_ *R_p_*, (μ_*xx*_ is the *xx* component of the viscosity tensor in Equation S29, Supporting Information) further verifying the correctness of our calculations. Therefore, the proposed light‐driven setup strongly boosts diffusion.

The radius *r_m_* reaches about half of the incident wavelength in water and thus the mechanical effect of optical vortices upon a nanoparticle takes place in the subwavelength region. Such a reduced scale cannot be reached using any focused far field, e.g., radial and Bessel beams.^[^ ^66^, ^67^, ^94^ ^]^ Up to our knowledge, this is the first proposal providing optical nanovortices created in a simple, realizable setup avoiding the need of lossy plasmonic nanoantennas,^[^ ^95^, ^96^ ^]^ short wavelength guided modes,^[^ ^97^ ^]^ or complex chiral structures.^[^ ^98^ ^]^ Such optical nanovortices represent a promising component for on‐a‐chip OAM exchange driving light−matter interactions (e.g., controlled light emission from quantum dots,^[^ ^98^ ^]^ superresolution,^[^ ^99^, ^100^ ^]^ and nano‐object manipulation^[^ ^66^, ^94^ ^]^).

## 5 Nanovortex‐Mediated Liquid Mixing

To study in detail the liquid flow driven by the proposed nanomixing design, we once again utilize direct time‐domain simulation in COMSOL Multiphysics. At each time step, the particle position and velocity, as well as the fluid pressure and velocity fields are obtained by solving Equation (7), the Navier−Stokes, and mass balance equations for the fluid.^[^ ^101^ ^]^ We consider simplified forms of the last two equations assuming laminar, incompressible flow, in accordance with the previous results for the particle trajectories. Furthermore, we impose open boundary conditions at the edges and simulate a large fluid domain around the nanocube (usually lab‐on‐a‐chip microchambers are of the order of tens of micrometers).

**Figure 7:**
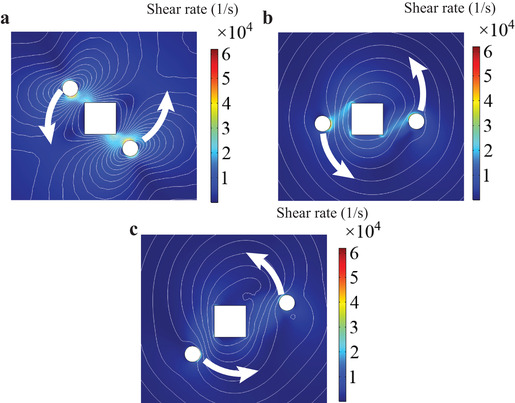
Formation of a fluid nanovortex due to the movement of two Au nanoparticles (white circles moving anticlockwise) driven by optical nanovortex formed around the nanocube (white square). Background color map denotes the distribution of stress in the fluid in 1 s^−1^ and white contours show the fluid velocity field streamlines of the initially static fluid at different times since the nanoparticles became optically driven. a) *t* = 0.01 µs, b) *t* = 150 µs and c) *t* = 260 µs. The represented domain has dimensions 800 × 800 nm. Parameters of the simulations are given in Table S1, Supporting Information. Open fluid boundary conditions were imposed at a distance of 1.5 µm from the center of the nanocube. For the sake of clarity, thermal motion is not taken into account in the simulation.

**Figure** **7** shows the calculated stresses and velocity fields in the fluid during 200 µs. The particles start with zero initial speed and gradually accelerate under the influence of radiation pressure and spin forces arising from their interaction with the optical nanovortex. Consequently, the fluid environment is also displaced, as Figure 7a demonstrates. At longer times, a single vortex‐like velocity distribution is established as shown in Figure 7c.

The velocity streamlines are more inhomogeneous at shorter times, when the nanoparticles start moving. Already at 150 µs, only small fluid distortions take place very close to the nanoparticles and the nanocube. Therefore, a possible way to further enhance the fluid nanomixing would be to realize periodic switching between left‐ and right‐hand circularly polarized incident light, which would reverse the direction of particle motion maintaining a high level of inhomogeneity in the fluid stress field.

Noteworthy that, while all the previous calculations were performed for Au nanoparticles in the visible range, similar dynamics can also be obtained for Ag nanoparticles in the UV range of the spectrum, where α ′ → 0.^[^ ^102^ ^]^ Nanomixing in the UV region could be advantageously combined with photochemically active processes of the involved chemical compounds.

## 6 Optical Sorting of Au Nanoparticles via the Nanovortex

Hereinafter, we demonstrate the important capability of the proposed configuration to realize optical force‐mediated particle on‐chip sorting. In this section, we illustrate a novel, dynamical, contact‐less size sorting method for Au nanoparticles in liquid solutions addressing one of the most challenging targets of conventional microfluidics with the help of dielectric nanophotonics.

**Figure 8:**
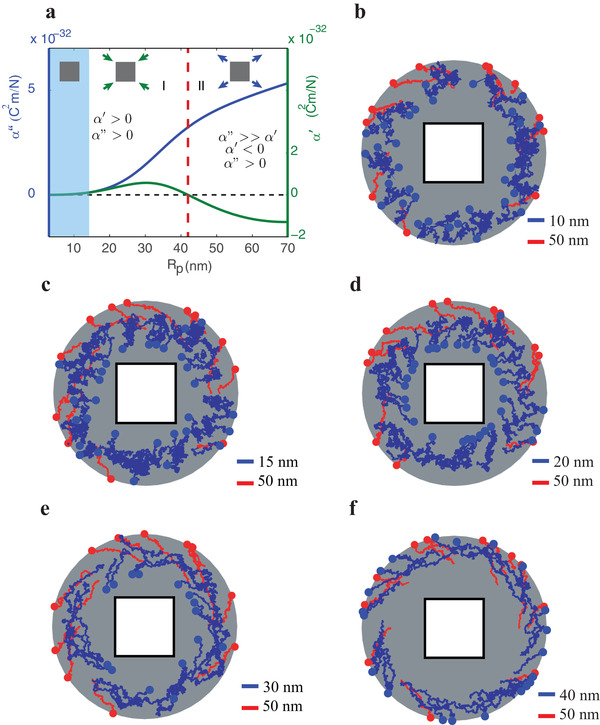
a) Real (α ′), and imaginary (α ′′) parts of the dipole polarizability as a function of the particle radius *R_p_* for an incident free space wavelength of 532 nm. The black dashed line indicates zero real or imaginary part, while the red dashed line shows the border between region I and II. The blue shaded area corresponds to the size region, where Brownian motion dominates. Insets depict schematically the radial direction of the optical forces exerted on Au nanoparticles inside the different regions. b−f) The blue curves show the calculated trajectories followed by particles with sizes from region I: 10 nm (b), 15 nm (c), 20 nm (d), 30 nm (e), and 40 nm (f), respectively. For comparison, in all the plots (b−f) the red curves correspond to the statistically different trajectories followed by nanoparticles with 50 nm radii corresponding to region II.

The proposed method is based on the sign switch displayed by α ′ close to the plasmon resonance as we demonstrated in Figure 4. The transition reverses the direction of the radial gradient force acting upon the nanoparticle (see the first term in Equation (5)). At a given incident wavelength, we can split the behavior of the Au nanoparticles into regions I and II (see **Figure** **8**a). Smaller nanoparticles from region I with positive α ′are attracted by the radial gradient force toward the nanocube, while larger nanoparticles, from region II, should be repelled outwards (see schematic insets in Figure 8a).

However, in region I, there is a competition between Brownian, gradient, and scattering forces, where the first introduces an unbiased random displacement, and the other two drag the nanoparticles in opposite radial directions. A careful analysis considering the contributions of each force is therefore required to determine the viability of the sorting method.

Figure 8b−f illustrates the proposed approach for nanoparticle separation by comparing the results of the numerical simulations for two sets of Au nanoparticles; the trajectories colored in blue correspond to nanoparticles with increasing size ranging from 10 to 40 nm radii, while the red trajectories in all Figure 8b−f are the paths followed by nanoparticles of 50 nm radius. The smaller particles in blue lie inside region I, while the larger red ones are in region II.

Nanoparticles smaller than 15 nm have very low polarizabilities, resulting in negligible optical forces in comparison with Brownian forces (Figure 8b). However, the numerical simulations show that nanoparticles with radii in the range 15–30 nm are drawn toward the nanocube tracing inward curved paths (see Figure 8d−f), and thus are primarily affected by gradient forces. The most intense inward forces in region I are experienced by nanoparticles with size 30 nm (Figure 8e) coinciding with the maximum of α ′ in Figure 8a, green line. Contrarily, nanoparticles with radii close to 40 nm (i.e., in the vicinity or inside region II), spiral away from the dielectric nanocube due to scattering forces. Larger particles are also repelled by the joint scattering and gradient forces, since the latter changes sign. As we have already mentioned, we performed additional numerical simulations proving the correctness of the dipole approximation for the larger nanoparticles (see Figures S3 and S4, Supporting information).

The results in this section demonstrate that our novel platform can operate as a sorting device for Au nanoparticles by exploiting their inward or outward motion toward the nanocube as a function of their dimension. A precise, in situ size control of Au nanoparticles is a crucial step in many applications, e.g., biological cell uptake rates,^[^ ^103^, ^104^ ^]^ toxicity,^[^ ^105^ ^]^ and Raman signal intensity.^[^ ^106^ ^]^

## 7 Conclusion

We present conditions for maximal conversion of SAM of the incident light to OAM of the scattered light via the specially designed transversely scattering silicon nanocube. The azimuthal component of the Poynting vector of the scattered field originates from the strong MQ resonance. A Au nanoparticle of appropriate size, illuminated by such optical field and dispersed in the fluid surrounding the nanocube, experiences a combination of spin and radiation pressure forces with non‐zero azimuthal component. They are significant only up to a distance of about half of the illuminating wavelength from the silicon nanoantenna. The exceptionally compact optical nanovortex drives the dynamics of the nanoparticles, inducing a convection fluid flow at the nanoscale. The direction of particle motion can be reversed simply by flipping the helicity of the incident circular polarization. Therefore, the proposed mechanism can serve as a nanoscale fluid mixer and diffusion booster driven by light in a contact‐less and flexible way. Arrays of the studied dielectric nanoantennae can be easily imprinted on the surface of a microfluidic chip and controllably illuminated in an independent fashion. Hence, we shed light on very exciting perspectives such as light‐controlled mixing or even on‐chip directional fluid navigation. Employing the dependence of the optical properties of Au nanoparticles on their size, we demonstrate feasibility to drag nanoparticles affected by the optical vortex either toward or outwards the nanocube. Thus, smaller nanoparticles (15−30 nm in radius) can be aggregated at the silicon nanoantenna surface, while larger nanoparticles move away from it and drive the fluid flow. This behavior can be utilized to perform in situ size separation directly inside the microfluidic chip.

The proposed, rather simple concept can be extended to nanostructures of different shapes, to an array of such nanostructures and to different types of dispersed nanoparticles, e.g., silver nanoparticles offer an exciting option to combine optical nanovortex with photochemistry at the nanoscale. Our approach opens a new room of opportunities for the integration of simple, optically driven nanosorting or filtering modules in on‐chip platforms paving the way toward more efficient functionalities in micro‐ and nanofluidic systems.

## Conflict of Interest

The authors declare no conflict of interest.

## Supporting information

Supporting Information
