# Supplementary material for: Nanovortex‐Driven All‐Dielectric Optical Diffusion Boosting and Sorting Concept for Lab‐on‐a‐Chip Platforms
Source: Adv Sci (Weinh). 2020 Apr 24;7(11):1903049. doi: 10.1002/advs.201903049 (PMC7284221; doi:10.1002/advs.201903049)
Supplement: Supplementary file 1 — Supporting Information [file ADVS-7-1903049-s001.pdf]

Copyright WILEY-VCH Verlag GmbH & Co. KGaA, 69469 Weinheim, Germany, 2020.

ADVANCED SCIENCE
Open Access

Supporting Information

for *Adv. Sci.,* DOI: 10.1002/advs.201903049

Nanovortex-Driven All-Dielectric Optical Diffusion Boosting and Sorting Concept for Lab-on-a-Chip Platforms

Adrià Canós Valero, Denis Kislov, Egor A. Gurvitz, Hadi K. Shamkhi, Alexander A. Pavlov, Dmitrii Redka, Sergey Yankin, Pavel Zemánek, and Alexander S. Shalin**

Supplemental Information

Nanovortex-driven all-dielectric optical diffusion boosting and sorting concept for lab-on-a-chip platforms

Adrià Canós Valero, Denis Kislov, Egor A. Gurvitz, Hadi K. Shamkhi, Alexander. A. Pavlov, Dmitrii Redka, Sergey Yankin, Pavel Zemánek and Alexander S. Shalin

1. Expressions for the total angular momentum of an electromagnetic field

In this first section we provide useful, well-known expressions regarding angular momentum which were utilized in the derivations of section 2 in the main text. The angular momentum of a circularly polarized plane wave can be written using the expressions for paraxial waves [1]. The z component can then be written in the general form

$$J_z(\mathbf{r}) = \frac{c\varepsilon_0}{2\omega}\left(\mathbf{E}_{inc}^* \cdot \mathbf{L}\mathbf{E}_{inc}\right)\Big|_z + 2c\left\langle \mathbf{L}_s \right\rangle\Big|_z, \tag{S1}$$

where $\mathbf{E}_{inc}$ is the electric field, ε_0 is the vacuum permittivity, $\mathbf{L}$ is the orbital angular momentum operator[2] defined explicitly in Equation (S15) , and $\left\langle \mathbf{L}_s \right\rangle = \varepsilon_0 n_m^2 \mathbf{E}\times\mathbf{E}^* / \left(4i\omega\right)$ is the electric contribution to the SAM flux density[3] of the beam in a medium with refractive index n_m. The first term in the right-hand side of (S1) corresponds to the OAM carried by $\mathbf{E}_{inc}$.

Additionally, without any loss of generality, the total angular momentum surface density of the field scattered by an object can be expressed as [2]:

$$\left\langle \mathbf{J} \right\rangle = \frac{\mathbf{r}\times\left\langle \mathbf{S}^s \right\rangle}{c}, \tag{S2}$$

where $\left\langle \mathbf{S}^s \right\rangle$ denotes the time-averaged scattered Poynting vector, and $\mathbf{r}$ is the position vector.

2. Excited components of the magnetic quadrupole under circular plane wave illumination

We now proceed to a rigorous derivation of Equations (2)-(3) of the main text. For that purpose, we now express the amount of power extracted from the field by the nanocube (referred to as the extinction power) [2] as

$$P_{ext} = \frac{1}{2}\mathrm{Re}\left\{\int_{V_p} \mathbf{E}_{inc}^* \cdot \mathbf{j}(\mathbf{r}) d^3\mathbf{r}\right\}, \tag{S3}$$

where the integration is carried inside the volume of the nanocube V_p of the nanocube, and $\mathbf{j}(\mathbf{r})$ is the induced current. We consider $\mathbf{E}_{inc}$ to be a circularly polarized plane wave with the form $\mathbf{E}_{inc}(\mathbf{r}) = \tilde{E}_0(z)\left(\mathbf{e}_x + i\sigma\mathbf{e}_y\right)$, with $\tilde{E}_0(z) = E_0 e^{-ik_0 z}$ and σ the helicity or spin of the incident

field. Following the steps outlined in Ref. [4], we consider the current inside the scatterer is primarily driven by the magnetic quadrupole, and therefore has the functional form

$$j_i(\mathbf{r}) \approx -\frac{1}{2}\varepsilon_{i\gamma\alpha}\partial_\gamma(M_{\beta\alpha}\partial_\beta\delta(\mathbf{r})), \qquad \text{(S4)}$$

in the previous, Einstein's summation convention has been adopted. j_i denotes the *ith* component of the internal current, $\varepsilon_{i\gamma\alpha}$ is the Levi-Civita symbol, ∂_γ is the spatial derivative over the component x_γ, $M_{\beta\alpha}$ is the $\beta\gamma$th component of the irreducible cartesian tensor defining the magnetic quadrupole moment and $\delta(\mathbf{r})$ is the 3-dimensional Dirac delta function. Inserting $\mathbf{E}_{inc}$ and Equation (S4) into (S3) allows to get rid of the volume integral since it cancels out due to the delta function. One obtains:

$$P_{ext} = -\frac{E_0 k_0^2}{4}\mathrm{Re}\left\{i\sigma M_{zx} + M_{zy}\right\} \qquad \text{(S5)}$$

Due to the symmetry of the nanocube, we note that both components must possess the same amplitude, so they can be written as $M_{zx} = ae^{i\phi_1}$ and $M_{zy} = ae^{i\phi_2}$, where $a = |M_{zx}|$. We also expect the phase delay between both components to be $\phi_2 - \phi_1 = \pm\pi/2$ since the x and y components of the incident electric field are also in this relation. Expanding the real part in the rhs of Equation (S5) we get

$$\mathrm{Re}\left\{i\sigma M_{zx} + M_{zy}\right\} = a\left(\sigma\cos\left(\phi_1 + \pi/2\right) + \cos\left(\phi_2\right)\right) \qquad \text{(S6)}$$

Considering the previous discussion, the only possible solution yielding non-zero contribution to extinction is

$$\phi_2 = \phi_1 + \sigma\frac{\pi}{2}, \qquad \text{(S7)}$$

which yields the exact relation between the excited components of the magnetic quadrupole:

$$M_{zy} = i\sigma M_{zx} \qquad \text{(S8)}$$

3. Higher order multipolar near-field vortices: magnetic quadrupoles

We now show a rigorous analytical proof illustrating the direct link between the excited magnetic quadrupole and the singular optical force vortex induced in the near field of the nanocube. We then make a comparison between the near fields of the optimized magnetic quadrupole nanocubes in two different host environments: liquid water (the one utilized in the main text) and air. In the latter case, an even more pronounced magnetic quadrupolar response can be obtained.

We start with the analysis of the multipole fields. In Ref.[5], the authors clearly showed the equivalence between the multipolar expansions in the Cartesian and Spherical representations. While the first one allows a more straightforward physical interpretation of the results, the second one can be more convenient for calculations. In what follows, we will make use of the

spherical representation. It is thus necessary for completeness to introduce the relations between the excited components of the Cartesian and Spherical 2nd rank tensors in our system, given in e.g. Ref. [6]:

$$\begin{aligned} T_{2-1} &\propto T_{xz} + iT_{yz} \\ T_{21} &\propto T_{xz} - iT_{yz} \end{aligned} \tag{S9}$$

with the usual restrictions:

$$T_{xx} + T_{yy} + T_{zz} = 0 \tag{S10}$$

and

$$T_{ij} = T_{ji} \tag{S11}$$

T_{ij} is the ijth component of a Cartesian tensor representing, e.g., the magnetic quadrupole. A component of a spherical tensor is also represented by two integer indices in the form T_{lm}, where $l = 1, 2, ...$ is the order of the multipole (dipole, quadrupole, and so on) and $m = -l, ..., l$ is proportional to the z component of angular momentum J_z carried by the multipole field[6]. Under circular plane wave illumination propagating along $-z$ and helicity σ, the symmetry of the nanocube in the x and y directions restrict the excited electric and magnetic quadrupole components to

$$T_{xz} + i\sigma T_{yz} = 0 \tag{S12}$$

Substituting Equation (S12) into (S9) it is straightforward to show that the only excited spherical multipole component will be of the form $T_{2\sigma}$, i.e. $m = \sigma$. This is in agreement with angular momentum conservation[6].

Neglecting reflections from the glass substrate, the scattered electric fields outside an arbitrary confined source under plane wave illumination with amplitude E_0 can be written as a superposition of vector spherical harmonics[7]:

$$\begin{aligned} \mathbf{E}_s(r,\theta,\phi) = E_0 \sum_{l=1}^{\infty} \sum_{m=-l}^{l} \mathrm{i}^l [\pi(2l+1)]^{1/2} \Big\{ \frac{1}{k} a_{lm} \nabla \times \left[h_l^{(1)}(kr) \mathbf{X}_{lm}(\theta,\phi) \right] \\ + b_{lm} h_l^{(1)}(kr) \mathbf{X}_{lm}(\theta,\phi) \Big\} \end{aligned} \tag{S13}$$

where the electric and magnetic multipoles are expressed in the spherical basis through the a_{lm}, b_{lm} coefficients, respectively, k is the wavevector in the host medium, $h_l^{(1)}(kr)$ are the first order spherical Hankel functions and the vector spherical harmonics $\mathbf{X}_{lm}(\theta,\phi)$ have the form

$$\mathbf{X}_{lm}(\theta,\phi) = \frac{1}{\sqrt{l(l+1)}} \mathbf{L} Y_{lm}(\theta,\phi) \tag{S14}$$

with $Y_{lm}(\theta,\phi)$ the spherical harmonic functions. The orbital angular momentum operator can be expressed in spherical coordinates:

$$\mathbf{L} = -i\left(0, -\frac{1}{\sin\theta}\frac{\partial}{\partial\phi}, \frac{\partial}{\partial\theta}\right) \tag{S15}$$

The total field outside the source is then the sum of the incident and scattered fields $\mathbf{E} = \mathbf{E}_s + \mathbf{E}_{inc}$. Nevertheless, since we are interested in the near and mid-range fields, in a first approach we can approximate the total field as $\mathbf{E} \approx \mathbf{E}_s$. The total scattering forces (considering both spin and Poynting vector contributions) exerted on a dipolar particle by an arbitrary field can be grouped together in[3]

$$\mathbf{F}_{sca} = \frac{\alpha''}{2}\mathrm{Im}\left\{(\nabla\mathbf{E})^T \cdot \mathbf{E}^*\right\} \tag{S16}$$

We have now all the necessary tools to investigate analytically the near field forces of the nanocube presented in the main text. Specifically, by virtue of Equations (S9) and (S12) and discussions therein, the problem reduces to analyzing the behavior of the forces induced by a magnetic quadrupole source of the form $b_{2\sigma}$, since it corresponds to the resonant contribution to scattering from the nanocube. Setting $l = 2, m = \sigma$ for the magnetic coefficient in (S13) (corresponding to an incident circularly polarized wave) and considering all the other terms to be zero leads to a simplified expression for the magnetic quadrupole field

$$\mathbf{E}^\sigma(r,\theta,\phi) = -\sqrt{5\pi}E_0 b_{2\sigma} h_2^{(1)}(kr)\mathbf{X}_{2\sigma}(\theta,\phi), \tag{S17}$$

where $\sigma = \pm 1$for left- and right- hand polarization, respectively. Finally, substituting (S17) into (S16) we obtain the near field optical non-conservative forces exerted by a magnetic quadrupole to a dipolar particle. E.g. for $b_{2\sigma} = 1$ and $\alpha'' = 2$, the azimuthal component has the final closed expression

$$F_\phi^\sigma(k,r,\theta) = 25\pi\sigma\mathrm{E}_0^2 \frac{\left(k^4r^4 + 3k^2r^2 + 9\right)}{16k^6r^7}\sin\theta, \tag{S18}$$

confirming the vortex is confined in the near and mid-field regions. As demonstrated numerically in **Figure 3**, in the near-field of a multipole source, the azimuthal scattering forces are not necessarily proportional to the Poynting vector, since both spin and radiation pressure contributions have relevant roles. We can easily test this statement via Equation (S18). In fact, we note that at $z = 0$ $(\theta = \pi/2)$, the scattered azimuthal force has a maxima, while the azimuthal component of the scattered Poynting vector of the magnetic quadrupole (Equation (4) in the main text) is zero. This means that the optical force vortex is entirely driven by spin forces.

The resulting force fields of a left-handed magnetic quadrupole b_{21} are plotted in **Figure S1(a)**. Our analytical formulae are contrasted with the numerical results obtained for the nanocube, after a careful optimization of its size in order to maximize their magnetic quadrupole response

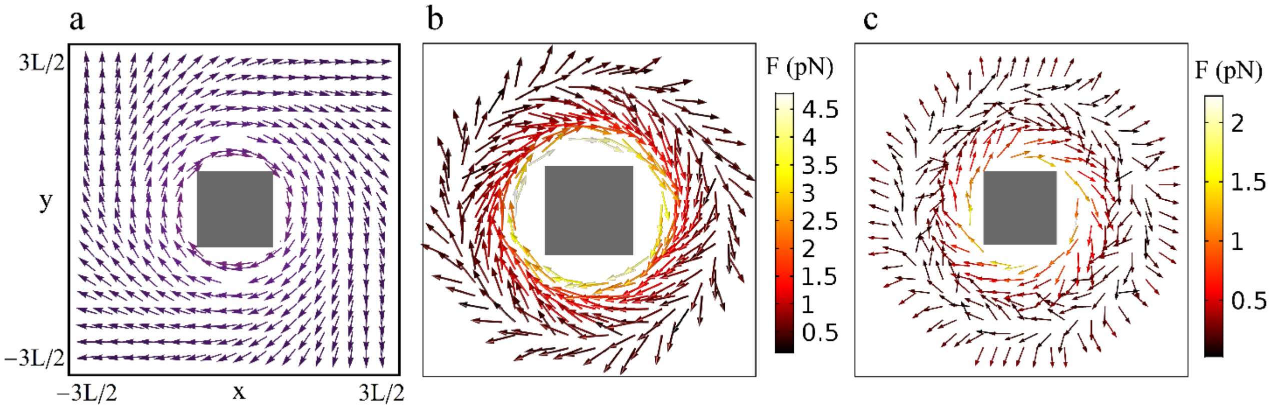

in air (**Figure S1(b)**) and water environments (**Figure S1(c)**).

Figure S1. Comparison between the analytical and numerical results for the optical forces at $z = 0$. (a) Normalized non-conservative forces exerted by the near-fields of a magnetic quadrupole b_{21} (induced by an incident LCP plane wave) calculated with Equations (S16)-(S17). Only the in-plane components are shown. The field is plotted far away from the singularity originating from the Hankel functions. L corresponds to the cube side. The cube has been drawn for illustration purposes. (b,c) Numerical calculations of the optimized magnetic quadrupole optical forces in two different host environments: (b) air ($n_m = 1$) and (c) water ($n_m = 1.335$). Incident power of 80 mW/μm^2. Note the cubes in air and water have different sizes, as a result of the optimization procedure for different environments. Their parameters are given in **Table S2**. Both (b,c) are plots in the near- and mid- field zones of the scatterer.

Interestingly, stronger azimuthal forces can be obtained in air. This is because the magnetic quadrupole resonance has a higher quality factor as a result of a larger refractive index contrast between the nanocube and air ~ 4, while in water the contrast is ~ 2.996. The multipole decomposition of the optimized nanocube in air is shown in **Figure S2**. The parasitic electric quadrupole contribution is also lower in this case. This can be appreciated by comparing **Figure S1(b,c)** with **Figure S1a**. The numerical results in air show a better defined vortex, while the optimized nanocube in water has small inward force components at some positions due to the electric quadrupole contribution. Nevertheless, the near field is still dominated by the magnetic quadrupole.

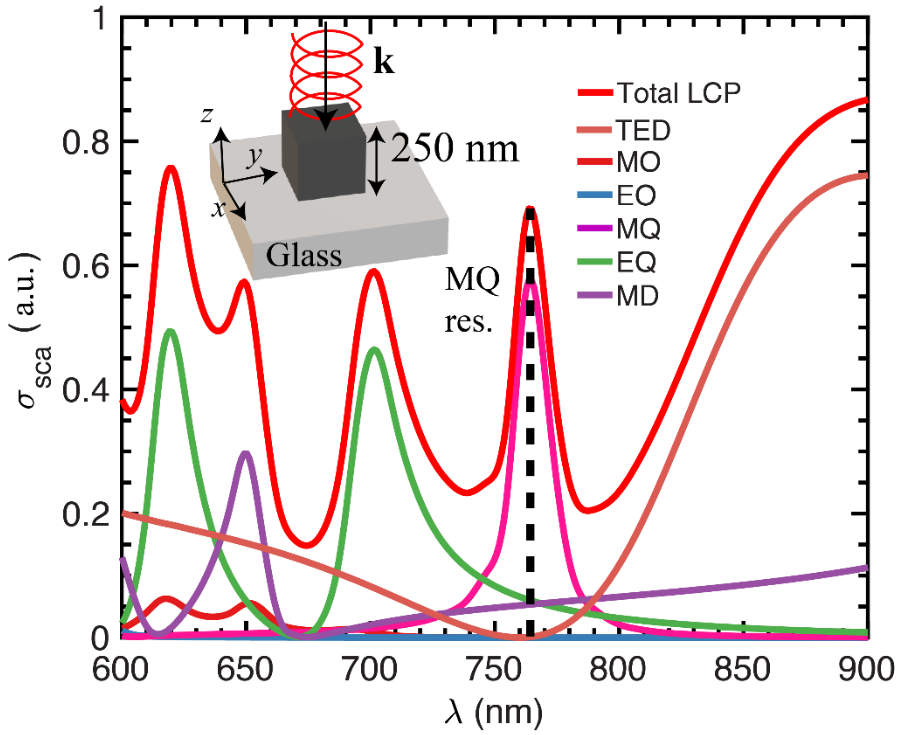

Figure S2. Cartesian multipole decomposition of the scattering cross-section of the silicon cube deposited on a glass substrate, centered at the origin of the coordinate system; the cube is illuminated by a left-hand circularly polarized plane wave propagating against the z-axis. The geometry is illustrated in the top inset and the ambient medium is air. The dashed black line indicates the position of the resonant MQ mode in this system (765 nm). The total scattered power with incident LCP illumination (Total LCP) is fully reconstructed as the sum of contributions of individual multipoles, respectively, the total electric dipole (TED), magnetic dipole (MD), electric quadrupole (EQ), and the magnetic quadrupole (MQ).

We remark that the considerations in this section are only valid when the influence of the substrate can be neglected. Otherwise, Equation (S13) must be modified in order to take into account the multipole reflections from the substrate. Unfortunately, no closed analytical solution exists for this problem, except for extreme cases when the substrate can be assumed a perfect electric or a perfect magnetic conductor[3].

4. Orbital torque induced by the scattering forces

A particle scattering as an electric dipole in an arbitrary electromagnetic field will be affected by an orbital torque entirely driven by the scattering forces. The z component of the optical torque Γ_z acting upon the nanoparticle due to $\langle \mathbf{F}_{sc} \rangle$ is found to be proportional to the tangential components of the Poynting vector $\langle S_\phi \rangle$ and the curl of $\langle \mathbf{L}_s \rangle$:

$$\Gamma_z = \left(\mathbf{r}\times\langle\mathbf{F}_{sc}\rangle\right)_z = r_\perp \frac{ck_0 n_m \alpha''}{\varepsilon_0}\left[\frac{1}{c^2}\langle S_\phi\rangle + \left(\nabla\times\langle\mathbf{L}_s\rangle\right)\Big|_\phi\right] \tag{S19}$$

with $r_\perp$ the distance to the z-axis. Equation (S19) clearly illustrates that the amount of orbital torque transmitted to the particles depends on the radiation pressure and the helicity spatial distribution of the scattered field by means of $\langle S_\phi\rangle$ and $\langle\mathbf{L}_s\rangle$, as well as the optical response of the particle itself by means of α''. In particular, Equation (S19) is utilized to obtain the results shown in **Figure 3**.

5. Dielectric function of Au nanoparticles

The calculations of the Au nanoparticle polarizabilities in **Figure 4** are performed for the well-known optical dispersion properties of bulk Au [8] (ε_b^{Au}), taking into account the Drude size corrections due to the limitation of the electron mean free path in small metallic particles [9]

$$\varepsilon_p^{Au}(\omega) = \varepsilon_b^{Au}(\omega) + \frac{\omega_{pl}^2}{\omega(\omega + i\gamma_b)} + \frac{\omega_{pl}^2}{\omega\left(\omega + i\gamma_b'\right)}\ , \tag{20}$$

$$\gamma_b' = \gamma_b + \frac{0.7 v_F}{R_p} \tag{21}$$

where ω_{pl}, γ_b and v_F are the plasma resonant frequency, the damping constant from the free electron Drude model, and the Fermi velocity, respectively.

6. Validity of the dipole approximation

The additional figures provided in this section are given in order to justify the validity of the dipole approximation considered in Equation.(9) for the analytical treatment of the optical forces induced by the dielectric scatterer in relatively large Au nanoparticles (20-50 nm radii).

In figure S1(A) we have plotted the exact contribution of electric (TM) and magnetic (TE) Mie coefficients to the total scattering cross section of a single 40 nm radius Au spherical nanoparticle submerged in water (i.e. environment refractive index is taken to be 1.34). The dispersion relation of Au is the same as in the main text. As it can be appreciated, radiation in the visible range is entirely described by the first electric Mie coefficient, corresponding to the total electric dipole contribution. Thus, this first result confirms that the investigated Au nanoparticles effectively radiate as dipoles. However, the derivation of Equation (9) additionally requires backscattering from the metallic nanoparticles to be negligible. Indeed, this is proven to be the case in figures S1(B-D), where the electric field distribution in the vicinity of the cube with and without the presence of 20 nm and 40 nm radii Au nanoparticles is shown for an illumination wavelength of 530 nm. The same parameters are utilized to demonstrate pure scattering force-driven spiral motion in section 4. The Au nanoparticle is placed at 150 nm distance from the

center of the cube, well inside the effective mixing radius r_m defined in section 4 and illustrated in figure 5. Indeed, only very localized field distortions can be observed even for the larger one. All field calculations have been performed by means of full 3D numerical analysis based on the finite element method implemented in Comsol Multiphysics[12].

Finally, we have also calculated the exact optical forces acting on the Au nanoparticles by straightforward integration of the Maxwell stress tensor over a spherical surface centered on the Au nanoparticle. The calculations confirm the presence of the tangential force distribution already observed with the help of Equation (9) (see figure S2). Very good agreement is observed in a direct comparison of the numerical solution with the analytical calculations. The validity of the dipole approximation for the studied size range is also confirmed by a number of other works [13–16].

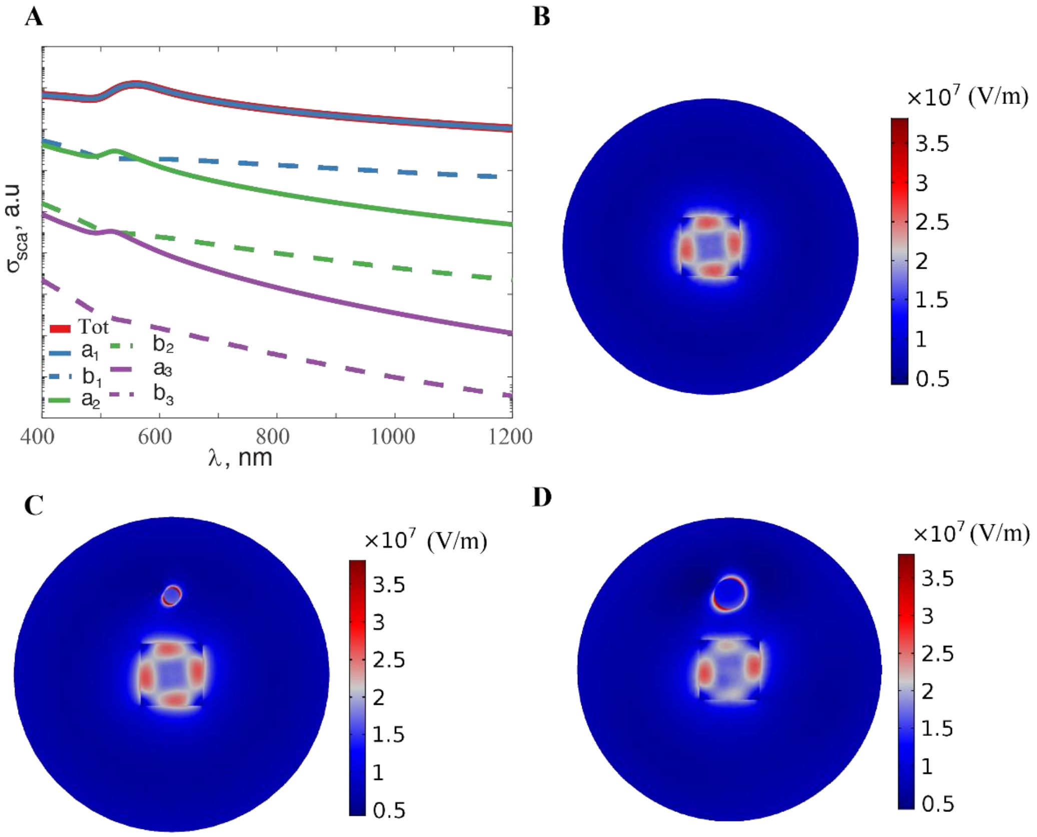

Figure S3. A) The contributions of the first 6 Mie coefficients to the total scattering cross section σ_{tot} of a single 40 nm radius Au nanoparticle submerged in water. B) Electric field norm in the vicinity of the dielectric cube in the absence of dipolar scatterers. C)-D) Electric field norm in the presence of 20 nm and 40 nm radius Au nanoparticles, respectively, placed at 200 nm from the center of the cube.

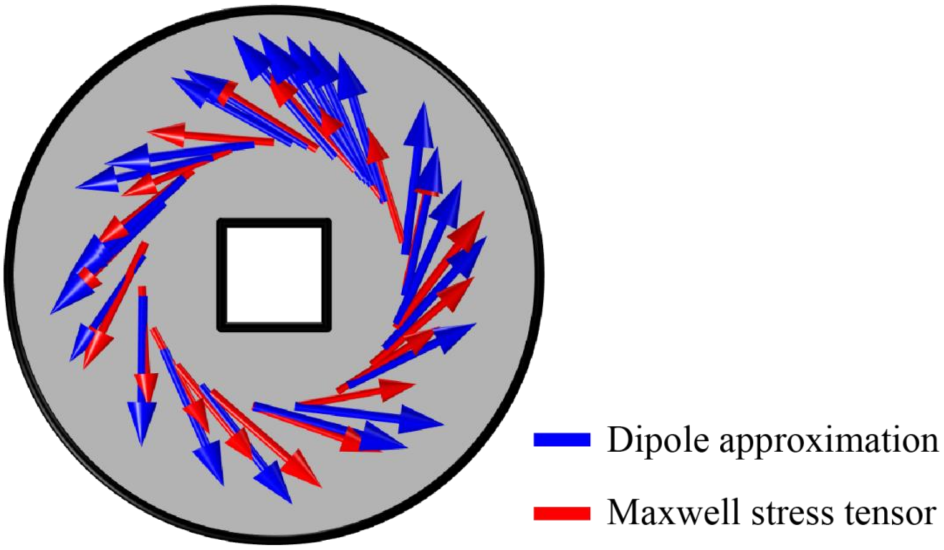

Figure S4. Red arrows: Optical force distribution numerically calculated by direct integration of the Maxwell stress tensor (COMSOL Multiphysics) at 200 nm from the center of the cube. The red arrow plot summarizes the results of a series of simulations where a 40 nm radius Au nanoparticle has been progressively displaced along the simulation domain. Each arrow corresponds to the optical force induced on the nanoparticle centered at its corresponding position in the plot. Blue arrows: optical forces calculated with the dipole approximation, (Equation (9) in the main text).

7. Simulation Parameters

Table S1. Information regarding materials involved in the hydrodynamical simulations.

Au	ρ_{Au} (kg/m^3)	ω_{pl} (rad/s) [17]	γ_b (rad/s) [17]	v_F (m/s) [17]	$\varepsilon_{Au}(\omega)$ (1)
	19300	1.297e16	1.52e14	1.4e6	Ref.[8]
Water	μ_0 (Pa s)	T (K)			
	8.9e-4	298			

Table S2. Geometry of the nanocube and resonant wavelengths of the magnetic quadrupole in different host environments.

Host medium†	Free Space ($n_h = 1$)	Water ($n_h = 1.335$)
Cube side (nm)	250	158
λ (nm) in vacuum	765	532

† All the simulations have been performed in the presence of a SCHOTT (N-BK7) glass substrate.

8. Analytical formulas taking into account particle-wall interactions: viscosity tensor

Accurate predictions of the particle dynamics require a quantitative understanding of particle-wall interactions. In a general situation the fluid flow around the nanoparticles will be affected by the walls of the nanocube, the glass substrate or the lateral walls of the microchamber. We

will take the first two into account in our model and neglect the effect of the third ones due to the reduced size of the optical nanovortex in comparison with the microchamber. Hereafter, we introduce the linearized Navier-Stokes equations and their solution in the presence of two perpendicular walls. Based on these formalism we are able to accurately reproduce the effective viscosity experienced by the nanoparticles in the cases of particle-substrate and particle-cube-substrate interactions.

We model the Au nanoparticles as point forces (Stokeslets) arising in the calm fluid due to the optical field and Brownian forces. In the viscous regime, the governing equations for the fluid motion are the linearized Navier-Stokes equations[18]

$$-\nabla p+\mu\nabla^2\mathbf{u}=-\mathbf{F}\delta\left(\mathbf{r}-\mathbf{r}_p\right) \quad \text{(S22)}$$

$$\nabla\cdot\mathbf{u}=0, \quad \text{(S23)}$$

where $\mathbf{F}$ is a point force corresponding to the external forces acting on a Au nanoparticle, $\mathbf{u}$ is the fluid velocity and p the pressure. We further impose $\mathbf{u}=0$ at infinity and at the positions of any wall in the system (no-slip). The general approach[19] consists in rewriting both $\mathbf{u}$ and p in terms of another vector function which we call $\boldsymbol{\psi}$:

$$\begin{aligned}\mathbf{u}&=\nabla\left(\mathbf{r}\cdot\boldsymbol{\psi}\right)-2\boldsymbol{\psi}\\ p&=2\mu_0\nabla\cdot\boldsymbol{\psi}\end{aligned}, \quad \text{(S24)}$$

each of the components are harmonic functions obeying the Laplace equation $\nabla^2\psi_n=0$, $n=1,2,3$. The boundary conditions can also be rewritten accordingly. Then the problem is separated in terms of the solution in an open homogeneous medium $\psi_n^{(1)}$, which is well-known[20], and the unknown term fulfilling the boundary conditions in the walls $\psi_n^{(2)}$ such that $\psi_n=\psi_n^{(1)}+\psi_n^{(2)}$. In our coordinate system, the general solution to the problem with two bounding walls is obtained after the system has been solved for the Fourier transform with respect to y and the Kontorovich-Lebedev transform[21] with respect to the variable $r_\perp=\sqrt{x^2+z^2}$, $\tilde{\psi}_n^{(2)}$. Alas [18]:

$$\psi_n^{(2)}(r,\theta,z)=\frac{1}{\pi^3}\int_{-\infty}^{\infty}e^{-isy}ds\int_0^{\infty}\nu\sinh(\pi\nu)K_{i\nu}(|s|r_\perp)\tilde{\psi}_n^{(2)}(\nu,\theta,s)d\nu, \quad \text{(S25)}$$

where the inverse Fourier and Kontorovich-Lebedev transforms have been applied, and $K_{i\nu}(|s|r_\perp)$ is the modified Bessel function of imaginary order. These integrals have been recently solved via a contour integration technique exploiting the residue theorem[18], and

previously numerical approximations were given in[19]. The expression for the drag force experienced by the Au nanoparticle in the vicinity of the nanocube can then be rewritten as[20]

$$\mathbf{F}_D = -6\pi R_p \bar{\bar{\mu}}(\lambda, q) \cdot \dot{\mathbf{r}}_p, \tag{S26}$$

with the parameter $q = R_p / |z_p|$ and λ is the ratio of the distance between the closest wall of the nanocube d and the radius of the nanoparticle R_p (see **Figure S3**). The effective viscosity $\bar{\bar{\mu}}(\lambda, q)$ is now a tensor quantity no longer corresponding to the viscosity of water, defined as:

$$\bar{\bar{\mu}}(\lambda, q) = \mu_0 \left[I - \bar{\bar{\mathrm{M}}}(\lambda) q + O\left(q^2\right) \right]^{-1} \tag{S27}$$

I is the identity matrix and the matrix $\bar{\bar{\mathrm{M}}}(\lambda)$ is

$$\bar{\bar{\mathrm{M}}}(\lambda) = \begin{pmatrix} k_1(\lambda) & 0 & k_2(\lambda) \\ 0 & k_3(\lambda) & 0 \\ k_2(\lambda) & 0 & k_1(1/\lambda) \end{pmatrix} \tag{S28}$$

The k_i functions are directly related to the integrals of Equation (S25). In our approach we simply interpolate their numerical values which are given in Table I and Table II of Ref.[19]. When the nanoparticle is sufficiently far away from the nanocube so that it only feels the effect of the glass substrate, $k_1(\lambda >> 1) = 9/16$, $k_2(\lambda >> 1) = 0$, $k_3(\lambda >> 1) = 9/16$, and $k_1(1/\lambda << 1) = 9/8$, recovering the well-known Faxen corrections in the presence of a plane wall[22–25].

In our simulations, we made sure that the centers of the nanoparticles were placed at a minimum separation of $d = 3R_p$ from the edges of the nanocube, to avoid excessive interactions. At $\lambda = 4$ ($d = 4R_p$), we already obtain $k_1 = 1.12629 \approx 9/8$, $k_2 = 0.00482$, $k_3 = 0.5762 \approx 9/16$, thus clearly showing that the effect of the walls of the nanocube is already negligible at this distance, and the glass substrate is the leading contribution to the effective viscosity tensor. Since $q \approx 1$ (i.e. the nanoparticles are almost in contact with the glass substrate due to radiation pressure in the z direction, as explained in the main text), they are at the onset of the lubrication regime[26]. We take this into account quantitatively by adding higher order corrections to $\bar{\bar{\mu}}(\lambda, q)$ [26,27]:

$$\bar{\bar{\mu}}(\lambda, q) = \mu_0 \left[I - \bar{\bar{\mathrm{M}}}(\lambda) q + H(\lambda \geq 4) \bar{\bar{N}} q^3 + O\left(q^5\right) \right]^{-1} \tag{S29}$$

$$\bar{\bar{N}} = \begin{pmatrix} 1/8 & 0 & 0 \\ 0 & 1/8 & 0 \\ 0 & 0 & 1/2 \end{pmatrix} \tag{S30}$$

Where $H(\lambda \geq 4)$ is a step function. Equation (S29) is the final expression for the viscosity tensor, encompassing the influence of the substrate and the walls of the nanocube into the drag forces acting on the Au nanoparticles. Interestingly, we note from the expression of $\bar{\bar{\mathrm{M}}}(\lambda)$ that nanoparticles may experience a small lift force when moving perpendicular to the cube. However, this force is orders of magnitude lower than the conventional drag force[19], and consequently it is fully compensated by the radiation pressure of the incident beam, keeping the movement restricted in the x-y plane.

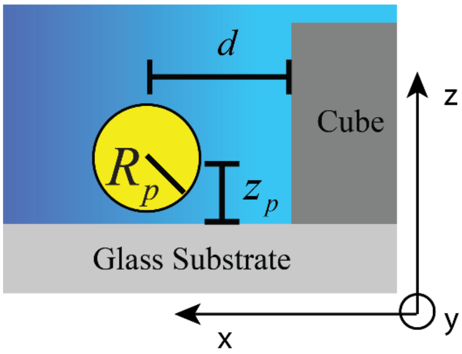

Figure S5. Geometry of the problem. The drag experienced by a Au nanoparticle is mainly affected by the glass substrate since $|z_p| \approx R_p$, but small separations d from the nanocube may also induce additional contributions to the effective viscosity tensor. All these interactions are included in Equation (S29).

9. Expressions for viscous and Brownian forces in the laminar regime

For small spherical geometries, the Brownian and viscous forces can be expressed as [10]:

$$\mathbf{F}_B = \mathbf{\Phi} \cdot \sqrt{\frac{12\pi k_B T}{\tau_c} R_p \bar{\bar{\mu}}(\lambda, q)} \qquad \text{(S31)}$$

$$\mathbf{F}_D = -6\pi R_p \bar{\bar{\mu}}(\lambda, q) \cdot \dot{\mathbf{r}}_p \qquad \text{(S32)}$$

where $\bar{\bar{\mu}}(\lambda, q)$ is the effective dynamic viscosity tensor in water including corrections taking into account the effect of the substrate and the nearest wall of the nanocube on the fluid flow given by Equation (S29), $\mathbf{\Phi}$ is a dimensionless vector function of randomly distributed numbers with zero mean [10], T is the temperature of the system, k_B is Boltzmann's constant and $\tau_c \approx 3\text{ns}$ is the momentum relaxation time for Au nanoparticles in water[11]. With the help of Equations **Error! Reference source not found.-Error! Reference source not found.**, Equation (8) of the main text can be solved in the Comsol Multiphysics© software package utilizing its particle tracing functionality.

10. Calculations of the MSAD and MSD

The MSAD and MSD shown in the main text were calculated by averaging the azimuthal angle and the particle position as a function of time for $N = 200$ Au nanoparticles with $R_p = 40\,\text{nm}$ [28]:

$$\left\langle \phi(t)^2 \right\rangle = \frac{1}{N}\sum_{i=1}^{N} \left| \phi_i(t) - \phi_i(0) \right|^2 \tag{S33}$$

$$\left\langle r(t)^2 \right\rangle = \frac{1}{N}\sum_{i=1}^{N} \left| \left(x_i(t) - x_i(0)\right)^2 + \left(y_i(t) - y_i(0)\right)^2 \right| \tag{S34}$$

The Einstein-Smoluchowski relation then tells us that, for times larger than the momentum relaxation time, Equation (S34) can be written as $\left\langle r(t)^2 \right\rangle = 4D_0 t$ if no external force acts on the system. D_0 is the diffusion coefficient of the Au nanoparticles in water, which can be approximated as $D_0(\lambda, q) = k_B T / 6\pi\mu_{xx}(\lambda, q) R_p$, where $\mu_{xx}(\lambda, q)$ is the xx component in Equation (S29). Similarly, the MSAD can be written as $\left\langle \phi(t)^2 \right\rangle \approx D_R^{eff} t$ in the limit [11] $D_R^{eff} t << 1$, where D_R^{eff} is an effective rotational diffusion coefficient with respect to the center of the nanocube. Under the influence of the non-conservative optical forces, anomalous diffusion takes place, modifying the Einstein relations to $\left\langle r(t)^2 \right\rangle = 4D_0 t + v_{avg}^2 t^2$ and $\left\langle \phi(t)^2 \right\rangle \approx D_R^{eff} t + \omega_{avg}^2 t^2$, where now v_{avg} and ω_{avg} are the average speed and angular velocities of the nanoparticles propulsed in the near field of the nanocube.

References

1. Dogariu, A. & Schwartz, C. Conservation of angular momentum of light in single scattering. *Opt. Express* **14**, 8425 (2006).
2. Jackson, J. D. *Classical electrodynamics*. (Wiley, 1999).
3. Novotny, L. & Hecht, B. *Principles of Nano-Optics*. (Cambridge University Press, 2012). doi:10.1017/CBO9780511794193
4. Evlyukhin, A. B., Fischer, T., Reinhardt, C. & Chichkov, B. N. Optical theorem and multipole scattering of light by arbitrarily shaped nanoparticles. *Phys. Rev. B* **94**, 1–7 (2016).
5. Alaee, R., Rockstuhl, C. & Fernandez-Corbaton, I. An electromagnetic multipole expansion beyond the long-wavelength approximation. *Opt. Commun.* **407**, 17–21 (2018).
6. Jackson, J. D. *Classical electrodynamics*. (1999).

7. Grahn, P., Shevchenko, A. & Kaivola, M. Electromagnetic multipole theory for optical nanomaterials. *New J. Phys.* **14**, 0–11 (2012).

8. Johnson, P. B. & Christy, R. W. Optical constants of the noble metals. *Phys. Rev. B* **6**, 4370–4379 (1972).

9. Shalin, A. S. Microscopic theory of optical properties of composite media with chaotically distributed nanoparticles. *Quantum Electron.* **40**, 1004–1011 (2010).

10. Kim, M. M. & Zydney, A. L. Effect of electrostatic, hydrodynamic, and Brownian forces on particle trajectories and sieving in normal flow filtration. *J. Colloid Interface Sci.* **269**, 425–431 (2004).

11. Philipse, A. P. *Brownian Motion: Elements of Colloid Dynamics*. *Brownian Motion* (Springer International Publishing, 2018).

12. Roger W. Pryor, P. *Multiphysics Modeling Using COMSOL®: A First Principles Approach*. *System* (Jones & Bartlett Publishers, 2009).

13. Hoefelmeyer, J. D., Niesz, K., Somorjai, G. a & Tilley, T. D. Expanding the optical trapping range of gold nanoparticles. *Nano Lett.* **5**, 1937–1942 (2005).

14. Svoboda, K. & Block, S. M. Optical trapping of metallic Rayleigh particles. *Opt. Lett.* **19**, 930 (1994).

15. Albaladejo, S., Marqués, M. I., Scheffold, F. & Sáenz, J. J. Giant enhanced diffusion of gold nanoparticles in optical vortex fields. *Nano Lett.* **9**, 3527–3531 (2009).

16. Bogdanov, A. A., Shalin, A. S. & Ginzburg, P. Optical forces in nanorod metamaterial. *Sci. Rep.* **5**, 1–9 (2015).

17. Khlebtsov, N. G. Optics and biophotonics of nanoparticles with a plasmon resonance. *Quantum Electron.* **38**, 504–529 (2008).

18. Dauparas, J. & Lauga, E. Leading-order Stokes flows near a corner. *IMA J. Appl. Math.* **83**, 590–633 (2018).

19. Sano, O. & Hasimoto, H. Slow Motion of a Spherical Particle in a Viscous Fluid Bounded by Two Perpendicular Walls. *Journal of the Physical Society of Japan* **40**, 884–890 (1976).

20. Blake, J. R. A note on the image system for a stokeslet in a no-slip boundary. *Math. Proc. Cambridge Philos. Soc.* **70**, 303–310 (1971).

21. JONES, D. S. The Kontorovich-Lebedev Transform. *IMA J. Appl. Math.* **26**, 133–141 (1980).

22. Schäffet, E., Nørrelykke, S. F. & Howard, J. Surface forces and drag coefficients of microspheres near a plane surface measured with optical tweezers. *Langmuir* **23**, 3654–3665 (2007).

23. Vermeulen, K. C., Wuite, G. J. L., Stienen, G. J. M. & Schmidt, C. F. Optical trap stiffness in the presence and absence of spherical aberrations. *Appl. Opt.* **45**, 1812–1819 (2006).

24. Leach, J. *et al.* Comparison of Faxén's correction for a microsphere translating or rotating near a surface. *Phys. Rev. E - Stat. Nonlinear, Soft Matter Phys.* **79**, (2009).

25. Kim, S., Karrila, S. J. & Brenner, H. *Microhydrodynamics: Principles and Selected Applications*. (Elsevier Science, 2013).

26. Bian, X., Kim, C. & Karniadakis, G. E. 111 years of Brownian motion. *Soft Matter* **12**, 6331–6346 (2016).

27. Perkins, G. S. & Jones, R. B. Hydrodynamic interaction of a spherical particle with a planar boundary. *Phys. A Stat. Mech. its Appl.* **189**, 447–477 (1992).

28. Kharazmi, A. & Priezjev, N. V. Molecular Dynamics Simulations of the Rotational and Translational Diffusion of a Janus Rod-Shaped Nanoparticle. *J. Phys. Chem. B* **121**, 7133–7139 (2017).
